# Change in Weight Status From Childhood to Young Adulthood and Risk of Adult Coronary Heart Disease

**DOI:** 10.1001/jamapediatrics.2025.4950

**Published:** 2025-12-01

**Authors:** Claes Ohlsson, Rebecka Bramsved, Maria Bygdell, Jari Martikainen, Annika Rosengren, Jenny M. Kindblom

**Affiliations:** 1 Centre for Bone and Arthritis Research, Department of Internal Medicine and Clinical Nutrition, Institute of Medicine, Sahlgrenska Academy, University of Gothenburg, Gothenburg, Sweden; 2 Unit of Clinical Pharmacology, Department of Pharmaceuticals, Sahlgrenska University Hospital, Region Västra Götaland, Gothenburg, Sweden; 3 Department of Pediatrics, Institute of Clinical Sciences, Sahlgrenska Academy, University of Gothenburg, Gothenburg, Sweden; 4 Research and Development Primary Health Care Södra Älvsborg, Sollebrunn Health Centre, Region Västra Götaland, Borås, Sweden; 5 Bioinformatics and Data Centre, Sahlgrenska Academy, University of Gothenburg, Gothenburg, Sweden; 6 Department of Molecular and Clinical Medicine, Sahlgrenska Academy, University of Gothenburg, Gothenburg, Sweden

## Abstract

**Importance:**

Childhood overweight is associated with adult coronary heart disease (CHD); the extent to which this risk can be mitigated by remission of childhood overweight before young adulthood is not clear.

**Objective:**

To evaluate if remission of elevated childhood weight before young adulthood mitigates the risk of adult CHD.

**Design, Setting, and Participants:**

This population-based cohort study was conducted among individuals born between 1945 and 1968 in Gothenburg, Sweden, as a part of the BMI Epidemiology Study (BEST). Archived child and school health records were linked to national high-quality registers in November 2022. Data analysis was performed from 2024 to 2025.

**Exposures:**

Childhood (women at age 7 years and men at age 8 years) and young adult (women age 18 years and men age 20 years) overweight derived from weight and height measurements in school health records and at conscription.

**Main Outcomes and Measures:**

The primary outcome was register-derived CHD diagnosis (fatal or nonfatal) in adult age.

**Results:**

This study included 103 232 individuals (45 965 women [44.5%]; mean [SD] childhood body mass index, calculated as weight in kilograms divided by height in meters squared, of 15.6 [1.5]) born 1945-1968 in Gothenburg, Sweden. Childhood and young adult overweight were associated with increased risk of CHD in both men and women. No significant sex interaction was observed for these associations. Remission of childhood overweight before young adulthood resulted in a similar risk of CHD as in individuals who had persistent normal weight (reference category; hazard ratio [HR], 0.98; 95% CI, 0.84-1.14). Both pubertal onset overweight (ie, normal weight in childhood and overweight in young adulthood; HR, 1.83; 95% CI, 1.66-2.03) and persistent overweight (ie, overweight in both childhood and young adulthood; HR, 1.53; 95% CI, 1.30-1.78) were associated with increased risk of adult CHD events. However, individuals with pubertal onset overweight had higher risk of CHD than individuals with persistent overweight (HR, 1.23; 95% CI, 1.03-1.49; *P* = .03).

**Conclusions and Relevance:**

In this population-based cohort study, increased risk of CHD in Swedish individuals with childhood overweight was reversed with remission of overweight before young adulthood; furthermore, overweight in young adulthood with pubertal onset was associated with higher risk of adult CHD compared to overweight persistent throughout childhood and puberty. These findings have implications for public health planning, emphasizing the importance of early detection and treatment of overweight during childhood and adolescence.

## Key Points

**Question:**

Does remission of childhood overweight before young adulthood reduce the risk of adult coronary heart disease (CHD) observed for childhood overweight?

**Findings:**

In this population-based cohort study in 103 000 Swedish men and women, remission of childhood overweight before young adult age resulted in a similar risk of CHD as individuals who had normal weight in childhood and young adulthood.

**Meaning:**

Individuals with overweight in childhood who have normal weight in young adulthood have similar risk of CHD as individuals with normal weight in childhood and young adulthood.

## Teaser

This population-based cohort study evaluates if remission of elevated childhood weight before young adulthood mitigates the risk of adult coronary heart disease.

## Introduction

Coronary heart disease (CHD) remains the leading cause of mortality globally in both women and men, with rapidly increasing rates in low- and middle-income countries.^1^ Many high-income countries have experienced declining incidence of CHD during the last decades due to improved treatment of both acute coronary events and risk factors, as well as public health efforts to reduce smoking.^2^ However, a recent rise in age-standardized CHD death rates has been observed in parts of the US and Great Britain, raising concerns that the global obesity epidemic during the last 50 years might have offset the medical advances in treatment and prevention of CHD.^3^

Adult overweight and obesity aggravate almost all cardiovascular risk factors and are linked to increased risk of CHD.^4^ Elevated body mass index (BMI, calculated as weight in kilograms divided by height in meters squared) in childhood has been shown to be associated with increased risk of adult CHD in both men and women,^5,6^ but it is not known to what extent excess risk persists if overweight in childhood is reversed before young adulthood.^7^

The population-based BMI Epidemiology Study (BEST) Gothenburg collected data on measured height and weight during childhood and puberty until young adulthood in a large cohort of men and women, with information on adult CHD retrieved from high-quality Swedish nationwide registers. The aim of the present study was to evaluate to what extent remission of elevated childhood weight affects adult CHD risk. For this, information on both prepubertal and postpubertal BMI is needed.

## Methods

### Study Population

The present study is a part of the population-based BEST Gothenburg cohort, initiated with the overall aim to study the impact of BMI during childhood and puberty on adult diseases.^8^ Men and women with data available on childhood BMI and young adult BMI (N = 103 232) were included in the study and were followed up from the age of 22 years until censoring due to being registered with a CHD diagnosis, death, migration, or until December 31, 2021, whichever occurred first. Individuals who died, emigrated, or had a CHD diagnosis registered before the age of 22 years were excluded. eFigure 1 in Supplement 1 shows the inclusion of individuals in the present study. The 40 985 individuals that were not included due to lacking 1 or both BMI variables or because of death, emigration, or a CHD event before the age of 22 years were 28.4% of eligible individuals. The Strengthening the Reporting of Observational Studies in Epidemiology (STROBE) reporting guidelines were followed.

### Exposures

Childhood overweight and obesity were defined according to the cutoffs for sex and age defined by the International Obesity Task Force (IOTF).^9^ Young adult overweight was defined as BMI of 25 or greater to less than 30, and obesity was defined as BMI of 30 or higher. High BMI was defined as a BMI above the 85th percentile for age and sex.

### Ethics Committee Approval

The Ethics Committee of the University of Gothenburg, Sweden, approved the study and waived the need for consent (013-10).

### Outcomes

Information on CHD and fatal CHD was obtained by linking the dataset through the personal identity number to the National Patient Register, initiated in 1964 with full coverage in the Gothenburg region from 1972, and to the Cause of Death Register, which contains information on causes of deaths since 1961, covering the entire follow-up period of this study.^10,11^ Outcomes were defined according to the *International Classification of Diseases (ICD)* system (eTable 1 in Supplement 1). The outcome, CHD, was defined as the first CHD event (fatal and nonfatal) as primary diagnosis.

### Statistical Analysis

Given the eligibility criteria, the main variables were available for all individuals in the included cohort (childhood BMI, young adult BMI, birth year, country of birth, and outcome). Descriptive data are presented as mean and SD. Categorical variables are presented as numbers and percentages. Follow-up started at age 22 years. Cox proportional hazards regression was used to estimate hazard ratios (HR) and 95% confidence intervals for the association between exposures and events, with all analyses adjusted for birth year and country of birth unless otherwise stated. Analyses in the full cohort are additionally adjusted for sex. When adjustment for socioeconomic status was performed, it was defined as educational attainment at 45 years of age. We evaluated early and late CHD events, defined according to the age of the median case, as before or after 57.6 years of age or before or after 44 years of age, respectively. Possible interactions were evaluated by addition of an interaction term (the variables of interest multiplied with each other) in the Cox regression models (*P* < .05 for an interaction term was interpreted as a statistically significant interaction). In addition to HR, absolute risks were calculated. The assumption of proportional hazards was evaluated by visual inspection of Schoenfeld residual plots and through proportional hazards test. To account for competing risks of non-CHD mortality, we used the Fine and Gray model. Correlation analyses were performed to estimate Pearson *r*.

In sensitivity analyses, we evaluated CHD risk in individuals born in Sweden and with parents born in Sweden. We excluded individuals with cancer, diabetes (definitions in eTable 1 in Supplement 1), and procedure codes F, N, and V before the age of 22 years, and we performed analyses with adjustment for diabetes diagnosed in the National Patient Register before event or censoring. All analyses were performed in SPSS version 29.0.1.1 (SPSS Statistics) or in R version 4.5.1 (R Foundation).

## Results

This study included 103 232 individuals, (45 965 women [44.5%]; mean [SD] childhood BMI of 15.6 [1.5]) born 1945-1968 in Gothenburg, Sweden. The prevalence of overweight (including obesity) in childhood according to the age- and sex-specific cutoffs from IOTF^9^ among the 103 232 individuals was 8.7% for girls and 4.4% for boys. In young adulthood, 5.1% of women and 7.7% of men had a BMI of 25 or higher. During 3 897 423 person-years of follow-up until December 31, 2021, 4438 men and 1298 women were diagnosed with a first CHD event, and 763 men and 168 women had a fatal CHD event (Table 1). The correlation between childhood BMI and pubertal BMI change was marginal in both women (*r*_p_ = −0.05) and men (*r*_p_ = 0.06), suggesting that these 2 BMI variables may contribute nonoverlapping information as risk markers for CHD in both women and men. In contrast, the correlation between childhood BMI and young adult BMI was strong in both women (*r*_p_ = 0.61) and men (*r*_p_ = 0.61). Individuals who could not be included because they were lacking 1 or both of the developmental BMI measurements did not differ substantially regarding childhood and young adult BMI from the included individuals (eTable 2 in Supplement 1). However, the nonincluded women had a slightly higher prevalence of CHD events.

**Table 1.** Cohort Characteristics

Characteristic	No. (%)		
	Full cohort (N = 103 232)^a^	Women (n = 45 965)	Men (n = 57 267)
Childhood BMI, mean (SD)^b^	15.6 (1.5)	15.5 (1.6)	15.7 (1.4)
Young adult BMI, mean (SD)^c^	21.1 (2.5)	20.8 (2.4)	21.4 (2.6)
Pubertal BMI change, mean (SD)	5.5 (2.0)	5.2 (1.9)	5.7 (2.0)
Follow-up period after age 22 y, mean (SD), y	37.8 (10.3)	38.1 (10.4)	37.4 (10.4)
Country of birth^d^			
Sweden	88 986 (86.2)	39 447 (85.8)	49 539 (86.5)
Other	14 246 (13.8)	6518 (14.2)	7728 (13.5)
Education level at age 45 y			
Primary school	13 177 (12.8)	4427 (9.6)	8750 (15.3)
Upper secondary school	45 394 (44.0)	19 856 (43.2)	25 538 (44.6)
Higher education	42 512 (41.2)	20 786 (45.2)	21 726 (37.9)
Missing	2149 (2.1)	896 (1.9)	1253 (2.2)
Childhood weight status^e^			
Overweight, including obesity	6473 (6.3)	3981 (8.7)	2492 (4.4)
Obesity	830 (0.8)	547 (1.2)	283 (0.5)
Young adult weight status^f^			
Overweight, including obesity	6767 (6.6)	2350 (5.1)	4417 (7.7)
Obesity	784 (0.8)	250 (0.5)	534 (0.9)
Diabetes before event or censoring	5293 (5.4)	1986 (4.3)	3932 (6.9)
CHD events	5736 (5.6)	1298 (2.8)	4438 (7.7)
Fatal CHD	931 (0.9)	168 (0.4)	763 (1.3)

Abbreviations: BMI, body mass index (calculated as weight in kilograms divided by height in meters squared); CHD, coronary heart disease.

^a^ Cohort characteristics for 103 232 women and men born between 1945 and 1968 (45 965 women and 57 267 men), followed up for a mean (SD) period of 37.6 (10.7) years after the age of 22 years.

^b^ Childhood BMI at age 7 years for girls, age 8 years for boys.

^c^ Young adult BMI at age 18 years for women, and age 20 years for men.

^d^ Sweden if child and both parents were born in Sweden.

^e^ Childhood overweight and obesity calculated at age 7 years for girls and age 8 years for boys using International Obesity Task Force cutoffs.^12^

^f^ Young adult overweight and obesity calculated at age 18 years for women and 20 years for men using the BMI cutoffs of 25 and 30, respectively.

### Childhood and Young Adulthood Overweight, Including Obesity, and the Risk of CHD

**Figure 1.**
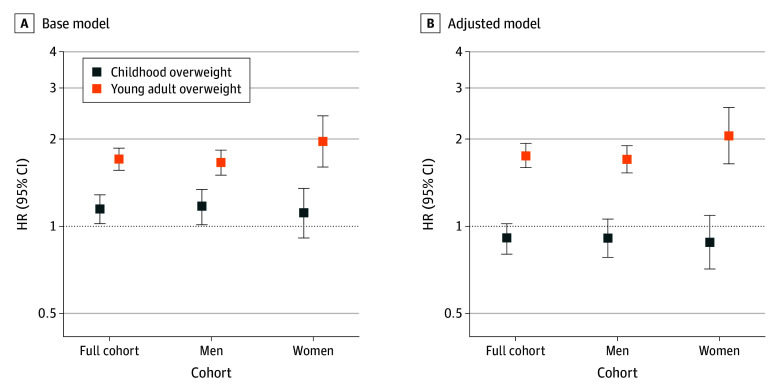
Association Between Childhood and Young Adult Overweight and the Risk of Coronary Heart Disease Events Hazard ratios (HRs, on log scale) estimated using Cox proportional hazards regression models with 95% confidence intervals and adjusted for birth year and country of birth; the analyses in the full cohort are additionally adjusted for sex. In the base model (A), HRs with 95% confidence intervals are presented for overweight in childhood compared to normal weight in childhood and for overweight in young adulthood compared to normal weight in young adulthood, respectively. In the adjusted models (B), the model for overweight in childhood is adjusted for overweight in adulthood, and the model for young adult overweight is adjusted for childhood overweight. Overweight (including obesity) in childhood was defined according to the International Obesity Task Force cutoffs for age and sex, and overweight (including obesity) in young adulthood was defined as a body mass index, calculated as weight in kilograms divided by height in meters squared, of ≥25.

Both childhood overweight (HR, 1.15; 95% CI, 1.02-1.28) and young adult overweight and obesity (HR, 1.71; 95% CI, 1.56-1.86) were directly associated with an increased risk of adult CHD events (Figure 1; eTable 3 in Supplement 1). No significant sex interaction was observed for these associations (sex × childhood overweight for CHD events: *P* = .67; for fatal CHD: *P* = .74; sex × young adult overweight for CHD events: *P* = .13; for fatal CHD: *P* = .07). However, when childhood overweight and young adult overweight were included in the same model, only young adult overweight was significantly associated with adult CHD (childhood overweight: HR, 0.91; 95% CI, 0.80-1.02; young adult overweight: HR, 1.75; 95% CI, 1.59-1.93) (Figure 1; eTable 3 in Supplement 1).

### Changes in Weight Status Between Childhood and Young Adulthood and the Risk of CHD

**Figure 2.**
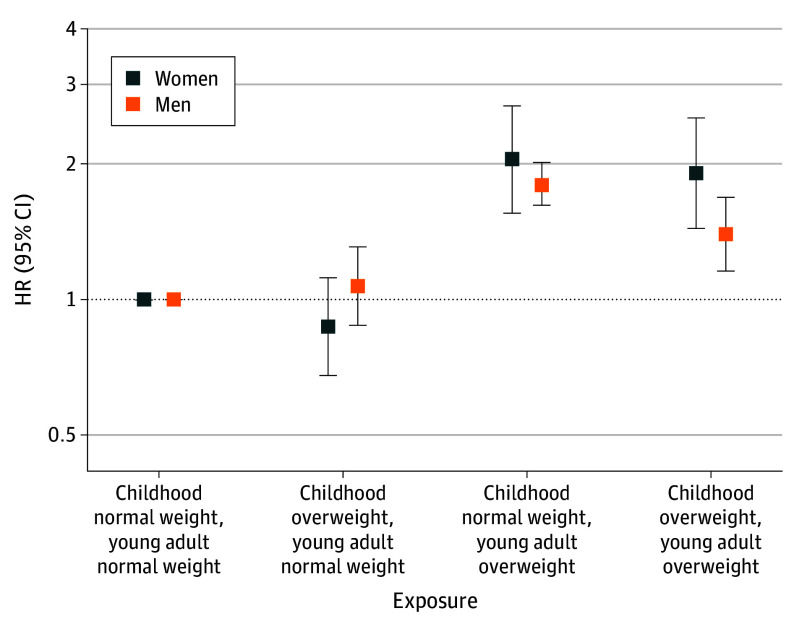
Risk of Coronary Heart Disease Events According to Overweight in Childhood and Young Adulthood Hazard ratios (HRs, on log scale) for overweight in childhood and young adult age, compared with normal weight in childhood and young adulthood, estimated using Cox proportional hazards regression models with 95% confidence intervals and adjusted for birth year and country of birth. HRs are estimated for the groups with overweight in childhood and normal weight in young adulthood, normal weight in childhood and overweight in young adulthood, and overweight in both childhood and young adulthood, with individuals with normal weight in childhood and young adulthood as the reference group. Overweight in childhood was defined according to the International Obesity Task Force cutoffs for age and sex, and overweight, including obesity, in young adulthood was defined as a body mass index, calculated as weight in kilograms divided by height in meters squared, of ≥25.

To examine the impact of changes in weight status from childhood to young adulthood for risk of CHD events in adulthood, we cross-categorized the cohort into 4 groups according to weight status in childhood and young adulthood. We used the group with normal weight in both childhood and young adulthood as the reference group (Figure 2; eTable 4 in Supplement 1). In this analysis, the 4091 individuals with remission of their childhood overweight before young adulthood (ie, childhood overweight and young adult normal weight) had a similar risk of CHD to individuals who never had been overweight (HR, 0.98; 95% CI, 0.84-1.14), indicating that the increased risk seen for individuals with overweight in childhood was reversible with remission of overweight before young adulthood. Sex-stratified analyses revealed similar results for men and women (Figure 2; eTable 4 in Supplement 1). Both pubertal onset overweight (ie, normal weight in childhood and overweight in young adulthood; HR, 1.83; 95% CI, 1.66-2.03) and persistent overweight (ie, overweight in both childhood and young adulthood; HR, 1.53; 95% CI, 1.30-1.78) were associated with an increased risk of adult CHD events (Figure 2; eTable 4 in Supplement 1). We evaluated the importance of pubertal onset overweight using a Cox regression model including only individuals with young adult overweight, with the group of individuals with persistent overweight as reference group. Of note, among the 6767 individuals with young adult overweight, those with pubertal onset overweight (ie, normal weight in childhood and overweight in young adulthood; n = 4385) had higher risk of adult CHD than individuals with persistent overweight (ie, overweight in both childhood and young adulthood; n = 2382; HR, 1.23; 95% CI, 1.03-1.49). This finding was mainly driven by the results in men (HR, 1.29; 95% CI, 1.04-1.59; women: HR, 1.07; 95% CI, 0.73-1.58). Descriptives for the overweight groups are presented in eTable 5 in Supplement 1. Adjustment for adult socioeconomic position resulted in similar findings (eTables 3 and 4 in Supplement 1).

We performed a number of sensitivity analyses. Similar results were obtained in all of them (eTables 6-8 in Supplement 1). Adjustment for socioeconomic status did not alter these results. Analyses of potential competing risks revealed similar results (eTable 9 in Supplement 1). We also evaluated early (before 57.6 years of age) and late (after 57.6 years of age) CHD (eTable 10 in Supplement 1), as well as CHD before and after 44 years of age (eTable 11 in Supplement 1).

In less powered analyses, we also evaluated changes in obesity status and the risk of CHD (see eResults and eFigure 3 in Supplement 1).

### BMI Percentile Changes Between Childhood and Young Adulthood and the Risk of CHD

The IOTF cutoffs used to define childhood overweight resulted in unbalanced proportions of overweight individuals according to sex and age (Table 1). To evaluate the risk of CHD in relation to high BMI with a balanced proportion of individuals with high BMI at both time points, we also performed analyses using the 85th percentile as the cutoff for high BMI and using the 15th percentile as the cutoff for low BMI. We found that individuals with a high childhood BMI (>85th percentile) and a normal (15th-85th percentiles) young adulthood BMI did not have higher risk of CHD than individuals with a normal BMI in both childhood and young adulthood (HR, 0.99; 95% CI, 0.90-1.10) (Table 2). In contrast, individuals with high young adulthood BMI (>85th percentile), regardless of BMI status in childhood, had an increased risk of adult CHD compared to individuals with a normal young adulthood BMI. The highest risk of adult CHD was observed for individuals who had a low childhood BMI (<15th percentile) and a high young adulthood BMI (>85th percentile) compared to individuals with a normal BMI both in childhood and in young adulthood (HR, 2.22; 95% CI, 1.53-3.22) (Table 2). Sex-stratified analyses revealed substantially similar results for men and women (Table 2), but an increased risk of CHD events was observed for women below the 15th percentile in childhood and with normal weight in young adulthood.

**Table 2.** Sex-Specific Body Mass Index (BMI) Percentile in Childhood by Sex-Specific BMI Percentile in Young Adulthood and the Risk of Coronary Heart Disease in Women and Men^a^

BMI percentile in childhood	BMI percentile in young adulthood					
	<15th		15th-85th		>85th	
	HR for CHD (95% CI)	No./CHD cases	HR for CHD (95% CI)	No./CHD cases	HR for CHD (95% CI)	No./CHD cases
**Full cohort**						
<15th	0.99 (0.89-1.10)	7154/407	1.07 (0.97-1.18)	8061/422	2.22 (1.53-3.22)	270/28
15th-85th	0.96 (0.87-1.05)	8159/477	1 [Reference]	56 274/2952	1.56 (1.42-1.71)	7829/549
>85th	1.03 (0.54-1.99)	172/9	0.99 (0.90-1.10)	7927/415	1.41 (1.28-1.56)	7386/477
**Women**						
<15th	1.02 (0.82-1.28)	3174/91	1.27 (1.04-1.56)	3638/112	2.86 (1.18-6.89)	83/5
15th-85th	1.07 (0.88-1.30)	3620/115	1 [Reference]	25 068/656	1.52 (1.25-1.85)	3487/116
>85th	NA	101/2	1.05 (0.85-1.30)	3469/96	1.37 (1.12-1.69)	3325/105
**Men**						
<15th	0.98 (0.87-1.10)	3980/316	1.01 (0.90-1.14)	4423/310	2.11 (1.40-3.18)	187/23
15th-85th	0.93 (0.83-1.04)	4539/362	1 [Reference]	31 206/2296	1.57 (1.41-1.74)	4342/433
>85th	1.18 (0.56-2.49)	71/7	0.98 (0.87-1.10)	4458/319	1.43 (1.28-1.59)	4061/372

Abbreviations: CHD, coronary heart disease; HR, hazard ratio; NA, not applicable.

^a^ HRs calculated using Cox proportional hazards regression with adjustment for birth year and country of birth, and for the full cohort additionally adjusted for sex. The full cohort included 103 232 women and men born between 1945 and 1968 (45 965 women and 57 267 men), followed up for a mean (SD) duration of 37.6 (10.7) years after the age of 22 years. In groups with <5 cases of CHD, no HR was calculated. Childhood BMI was calculated at ages 7 and 8 years for girls and boys, respectively, and young adult BMI was calculated at ages 18 and 20 years for women and men, respectively. Percentiles were calculated internal to this cohort.

We also performed analyses with the individuals cross-categorized in groups of normal or high childhood BMI and normal or high young adult BMI. The results of these analyses were similar (eResults and eTable 12 in Supplement 1). Thus, the results of these analyses using BMI percentile cutoffs for the definition of high BMI were similar to those using the conventional clinical overweight cutoffs.

Similar results were also seen in less powered analyses of the CHD risk in the group with a childhood or young adult BMI above the 95th percentile (eTable 13 in Supplement 1).

### Risk of Fatal Coronary Events

We also evaluated the risk of fatal CHD (eResults, eTable 12, and eFigure 2 in Supplement 1).

### Associations Between Continuous BMI Variables and the Risk of CHD

In a model with childhood BMI and pubertal BMI change included as continuous variables, pubertal BMI change was significantly associated with CHD events in the total cohort (HR, 1.19 per SD increase; 95% CI, 1.17-1.22), in women (HR, 1.21 per SD increase; 95% CI, 1.15-1.27), and in men (HR, 1.19 per SD increase; 95% CI, 1.16-1.22). In contrast, childhood BMI was not associated with CHD events in women or men. The associations between the pubertal BMI change and CHD events in the total cohort, women, and men were linear (full cohort: *P* = .09; men: *P* = .29; and women: *P* = .19 for pubertal BMI^2^). There was no significant interaction between baseline BMI and the pubertal BMI change (*P* > .99 for the interaction term childhood BMI × pubertal BMI change).

Expressed as excess risk per unit BMI increase, we found that 1 unit higher pubertal BMI change in women was associated with 10% excess risk of CHD events (HR, 1.10; 95% CI, 1.07-1.13) and 18% excess risk of fatal CHD (HR, 1.18; 95% CI, 1.11-1.26). For men, the corresponding excess risks for the pubertal BMI change were 9% and 12%, respectively (CHD events: HR, 1.09; 95% CI, 1.07-1.10; fatal CHD: HR, 1.12; 95% CI, 1.08-1.15).

## Discussion

Whether remission of childhood overweight before young adulthood alters the association between childhood overweight and increased risk of adult CHD has not been fully elucidated. In this large, population-based cohort study including 103 232 women and men with data reflecting BMI status in childhood (before puberty) and young adulthood (after puberty), we found that both childhood overweight (defined according to the IOTF cutoffs for age and sex) and young adult overweight (defined as a BMI >25) were associated with increased risk of adult CHD. Importantly, however, individuals with overweight in childhood who had normal weight in young adulthood did not differ in risk of adult CHD compared to individuals with normal weight in both childhood and young adulthood. These findings indicate that the increased risk seen for overweight in childhood is reversible with remission of overweight before young adulthood. Sex-stratified analyses revealed similar results separately in men and women. Individuals with pubertal onset overweight had higher risk of CHD compared to individuals with overweight throughout childhood and puberty. Our findings suggest that reversal of overweight and obesity in childhood resulting in remission before young adult age reduces the burden of CHD in adult age. These findings could have implications for preventive public health initiatives.

Several studies have reported associations between elevated BMI during the developmental years and increased risk of adult CHD,^6,13,14,15,16,17^ but these studies have not been able to evaluate the risk associated with overweight in childhood with remission before young adulthood. Our findings indicate that there is no excess risk of adult CHD in women or men with overweight (including obesity) in childhood with remission before young adult age, suggesting that, aside from general prevention measures, more active identification and treatment of childhood overweight is needed. Two recent studies^18,19^ evaluating the efficacy of anti-obesity behavioral treatment in children demonstrate that treatment initiated early in childhood has better chances of successful outcomes than treatment initiated later during development. Furthermore, a good treatment outcome before age 18 years was associated with a reduced risk of obesity-related complications in young adulthood.^20^ Based on the findings in the present study, we suggest that treatment should be initiated as soon as childhood overweight is identified.

Large epidemiological studies, as well as studies using the mendelian randomization approach, have demonstrated that overweight in young adulthood is associated with increased risk of adult CHD.^12,21,22^ This association has proven robust and independent of known confounding lifestyle factors, as well as BMI in midlife. However, these studies were not able to discriminate overweight in young adulthood with childhood onset from overweight with onset during the pubertal years. Childhood and puberty represent 2 physiologically distinct periods. We have previously demonstrated that the correlation between childhood BMI and the pubertal BMI change is very low in men. We herein show that the correlation between childhood BMI and pubertal BMI change is marginal not only in men, but also in women, suggesting that these 2 BMI variables may contribute nonoverlapping information as risk markers of adult CHD disease in both women and men.

In a subset of the BEST cohort including men born 1945-1961 (n = 37 672), we have previously demonstrated that pubertal BMI change, but not childhood BMI, was independently associated with cardiovascular disease and coronary atherosclerosis in men.^8,12,23,24^ In the present study using the expanded BEST cohort including both men and women born 1945-1968 (N = 103 232), we found that developing overweight during the pubertal years was associated with a higher risk of adult CHD compared to individuals who had persistent overweight from childhood into young adulthood. This finding is in line with the results from the ALSPAC study, where a high stable BMI between ages 7.5 and 24.5 years was associated with a more favorable cardiovascular risk profile than developing overweight after the age of 15.^25^ However, adult cardiovascular disease events were not yet available in the ALSPAC study. In a small Icelandic study, it was reported that a steep BMI increase between 8 and 13 years of age was associated with a high risk of fatal CHD.^25,26^ Thus, going from normal weight to overweight during puberty appears to be a particularly important factor involved in increasing the risk of adult CHD. This finding was not robust for obesity or fatal CHD, probably due to insufficiently powered analyses. To what extent findings in these individuals born 60 to 80 years ago are still applicable today is unknown, but global trends with respect to childhood obesity and premature cardiometabolic mortality are far from reassuring.

For women who were below the 15th percentile in childhood and with normal weight in young adult age, there was an increased risk of CHD events. This finding aligns with findings by Barker and colleagues^7^ that children who had coronary events as adults were smaller during early childhood years compared to those who did not have coronary events. However, we cannot completely rule out the possibility of reverse causality, that underweight is caused by disease that may also increase the risk of CHD—for example, an undiagnosed congenital heart disease.

Women experienced significantly fewer CHD events as well as fatal CHD compared to men, reflecting the fact that women develop atherosclerosis approximately 10 years later than men.^27^ However, there was no evidence of sex differences regarding the associations of childhood and young adulthood overweight with risk of adult CHD in the present study.

The observational design of the present study precludes causal conclusions. However, using a causal multivariable mendelian randomization approach, Power and colleagues^28^ provided evidence that childhood BMI mainly increases the risk of adult CHD indirectly via the pathway of adult body size. This is in line with the results from the present study.

### Strengths and Limitations

The strengths of this study include the well-powered population-based cohort design, with a near-complete follow-up of almost 40 years in both women and men, with data on both childhood and young adult BMI, as well as high-quality register-based diagnoses and causes of death. However, the study is not without limitations. We could not adjust for known lifestyle risk factors, such as smoking. In other studies evaluating the effects of young adult BMI, adjusting for smoking did not alter the results.^16,29^ Moreover, we cannot completely exclude residual confounding. As shown in eFigure 1 in Supplement 1, individuals with missing data on weight and height were not included in the study sample (28% of the eligible study population). Furthermore, the individuals in the cohort were predominantly White, and our conclusions might have limited generalizability with respect to other ethnicities. The rate of severe obesity was very low in the present cohort born 1945-1968. This study cannot, therefore, be conclusive regarding the risk estimates for individuals with severe obesity.

## Conclusions

In this population-based cohort study, results suggest that the increased risk of adult CHD associated with childhood overweight could be reversible with remission of overweight before young adulthood. In addition, developing overweight during the pubertal years was associated with a higher risk of adult CHD than overweight present already from childhood. Public health efforts aiming to prevent CHD in adults should include preventive measures in early life, as well as early treatment of childhood and pubertal overweight.

## Notes

Supplement 1. eMethods. Supplemental Methods eResults. Supplemental Results eTable 1. Diagnostic Codes According to the International Classification of Diseases (ICD) System Defining the Study Outcome Coronary Heart Disease (CHD) in the Patient Register and Cause of Death Register in Sweden eTable 2. Characteristics of Individuals Who Were Not Included eTable 3. Adjusted Hazard Ratios for Coronary Heart Disease (CHD) in Relation to Childhood and Young Adult Overweight, Including Obesity eTable 4. Absolute Risks and Adjusted Hazard Ratios for Coronary Heart Disease in Relation to Changes in Weight Status Between Childhood and Early Adulthood eTable 5. BMI in Childhood and Young Adulthood for Groups Categorized According to Normal Weight and Overweight Status in Childhood and Young Adult Age eTable 6. Absolute Risks and Adjusted Hazard Ratios for Coronary Heart Disease in Relation to Changes in Weight Status Between Childhood and Early Adulthood in Individuals With Both Parents Born in Sweden eTable 7. Absolute Risks and Adjusted Hazard Ratios for Coronary Heart Disease in Relation to Changes in Weight Status Between Childhood and Early Adulthood, After Exclusion of Individuals With Severe Disease or Major Surgery Before 22 Years of Age eTable 8. Adjusted Hazard Ratios for Coronary Heart Disease in Relation to Changes in Weight Status Between Childhood and Early Adulthood, With Adjustment for Diabetes Registered Before Event or Censoring eTable 9. Adjusted Hazard Ratios for Coronary Heart Disease in Relation to Changes in Weight Status Between Childhood and Early Adulthood, Accounting for Competing Risks of non-CHD Mortality Using the Fine and Gray Model eTable 10. Adjusted Hazard Ratios for Early (< 57.6) and Late (>57.6) Coronary Heart Disease in Relation to Changes in Weight Status Between Childhood and Early Adulthood eTable 11. Adjusted Hazard Ratios for Coronary Heart Disease Before and After 44 Years of Age, in Relation to Changes in Weight Status Between Childhood and Early Adulthood eTable 12. Absolute Risks and Adjusted Hazard Ratios for Coronary Heart Disease (CHD) Events and Fatal CHD, in Relation to Normal BMI (Under 85th Percentile) or High BMI (Over 85th Percentile) eTable 13. Adjusted Hazard Ratios for Coronary Heart Disease (CHD) Events in Relation to Sex-Specific BMI 95th Percentile in Childhood by Sex-Specific BMI 95th Percentile in Young Adulthood and the Risk of Coronary Heart Disease in Women and Men eFigure 1. Flowchart of Included Individuals in the Study eFigure 2. Risk of CHD Events and Fatal CHD According to BMI Above the 85th Percentile, in Childhood and Young Adulthood eFigure 3. Risk of CHD Events According to Obesity in Childhood and Young Adulthood eReferences.

Supplement 2. Data Sharing Statement
