# Supplementary material for: Change in Weight Status From Childhood to Young Adulthood and Risk of Adult Coronary Heart Disease
Source: JAMA Pediatr. 2025 Dec 1;180(2):179–86. doi: 10.1001/jamapediatrics.2025.4950 (PMC12670265; doi:10.1001/jamapediatrics.2025.4950)
Supplement: Supplement 1. — eMethods. Supplemental Methods eResults. Supplemental Results eTable 1. Diagnostic Codes According to the International Classification of Diseases (ICD) System Defining the Study Outcome Coronary Heart Disease (CHD) in the Patient Register and Cause of Death Register in Sweden eTable 2. Characteristics of Individuals Who Were Not Included eTable 3. Adjusted Hazard Ratios for Coronary Heart Disease (CHD) in Relation to Childhood and Young Adult Overweight, Including Obesity eTable 4. Absolute Risks and Adjusted Hazard Ratios for Coronary Heart Disease in Relation to Changes in Weight Status Between Childhood and Early Adulthood eTable 5. BMI in Childhood and Young Adulthood for Groups Categorized According to Normal Weight and Overweight Status in Childhood and Young Adult Age eTable 6. Absolute Risks and Adjusted Hazard Ratios for Coronary Heart Disease in Relation to Changes in Weight Status Between Childhood and Early Adulthood in Individuals With Both Parents Born in Sweden eTable 7. Absolute Risks and Adjusted Hazard Ratios for Coronary Heart Disease in Relation to Changes in Weight Status Between Childhood and Early Adulthood, After Exclusion of Individuals With Severe Disease or Major Surgery Before 22 Years of Age eTable 8. Adjusted Hazard Ratios for Coronary Heart Disease in Relation to Changes in Weight Status Between Childhood and Early Adulthood, With Adjustment for Diabetes Registered Before Event or Censoring eTable 9. Adjusted Hazard Ratios for Coronary Heart Disease in Relation to Changes in Weight Status Between Childhood and Early Adulthood, Accounting for Competing Risks of non-CHD Mortality Using the Fine and Gray Model eTable 10. Adjusted Hazard Ratios for Early (< 57.6) and Late (>57.6) Coronary Heart Disease in Relation to Changes in Weight Status Between Childhood and Early Adulthood eTable 11. Adjusted Hazard Ratios for Coronary Heart Disease Before and After 44 Years of Age, in Relation to Changes in Weight Status Between Childhood and Early [file jamapediatr-e254950-s001.pdf]

Supplemental Online Content

Ohlsson C, Bramsved R, Bygdell M, Martikainen J, Rosengren A, Kindblom JM. Change in weight status from childhood to young adulthood and risk of adult coronary heart disease. *JAMA Pediatr*. Published online December 1, 2025. doi:10.1001/jamapediatrics.2025.4950

eMethods. Supplemental Methods
eResults. Supplemental Results
eTable 1. Diagnostic Codes According to the *International Classification of Diseases* (*ICD*) System Defining the Study Outcome Coronary Heart Disease (CHD) in the Patient Register and Cause of Death Register in Sweden
eTable 2. Characteristics of Individuals Who Were Not Included
eTable 3. Adjusted Hazard Ratios for Coronary Heart Disease (CHD) in Relation to Childhood and Young Adult Overweight, Including Obesity
eTable 4. Absolute Risks and Adjusted Hazard Ratios for Coronary Heart Disease in Relation to Changes in Weight Status Between Childhood and Early Adulthood
eTable 5. BMI in Childhood and Young Adulthood for Groups Categorized According to Normal Weight and Overweight Status in Childhood and Young Adult Age
eTable 6. Absolute Risks and Adjusted Hazard Ratios for Coronary Heart Disease in Relation to Changes in Weight Status Between Childhood and Early Adulthood in Individuals With Both Parents Born in Sweden
eTable 7. Absolute Risks and Adjusted Hazard Ratios for Coronary Heart Disease in Relation to Changes in Weight Status Between Childhood and Early Adulthood, After Exclusion of Individuals With Severe Disease or Major Surgery Before 22 Years of Age
eTable 8. Adjusted Hazard Ratios for Coronary Heart Disease in Relation to Changes in Weight Status Between Childhood and Early Adulthood, With Adjustment for Diabetes Registered Before Event or Censoring
eTable 9. Adjusted Hazard Ratios for Coronary Heart Disease in Relation to Changes in Weight Status Between Childhood and Early Adulthood, Accounting for Competing Risks of non-CHD Mortality Using the Fine and Gray Model
eTable 10. Adjusted Hazard Ratios for Early (< 57.6) and Late (>57.6) Coronary Heart Disease in Relation to Changes in Weight Status Between Childhood and Early Adulthood
eTable 11. Adjusted Hazard Ratios for Coronary Heart Disease Before and After 44 Years of Age, in Relation to Changes in Weight Status Between Childhood and Early Adulthood
eTable 12. Absolute Risks and Adjusted Hazard Ratios for Coronary Heart Disease (CHD) Events and Fatal CHD, in Relation to Normal BMI (Under 85th Percentile) or High BMI (Over 85th Percentile)
eTable 13. Adjusted Hazard Ratios for Coronary Heart Disease (CHD) Events in Relation to Sex-Specific BMI 95th Percentile in Childhood by Sex-Specific BMI 95th Percentile in Young Adulthood and the Risk of Coronary Heart Disease in Women and Men
eFigure 1. Flowchart of Included Individuals in the Study
eFigure 2. Risk of CHD Events and Fatal CHD According to BMI Above the 85th Percentile, in Childhood and Young Adulthood
eFigure 3. Risk of CHD Events According to Obesity in Childhood and Young Adulthood
eReferences.

© 2025 Olsson C et al et al. *JAMA Pediatrics*.

This supplemental material has been provided by the authors to give readers additional information about their work.

© 2025 Olsson C et al et al. *JAMA Pediatrics*.

eMethods. Supplemental Methods

Exposures

Childhood BMI was calculated using all paired weight and height measurements between ages 6 and 8 years of age for girls, and 6.5 and 9.5 years of age for boys. Young adult BMI was calculated using measurements between 15.5 and 20.0 years of age for women (from school healthcare records) and 17.5 to 22.0 years for men (from school healthcare records and conscription). The BMI variables were age-adjusted for each individual participant using a linear regression model with BMI as the dependent variable and age as an independent variable. The age-dependent change was assumed to follow the slope of the fitted models. We estimated age-adjusted childhood BMI (at 7 years of age for girls and 8 years for boys) and young adult BMI (at 18 years of age for women and 20 years for men) using the slope of the fitted models. Pubertal BMI change was defined as the difference between young adult BMI and childhood BMI (for women BMI at age 18 minus BMI at age 7, and for men BMI at 20 years minus BMI at 8 years of age). For adjustment for socioeconomic status, we obtained information on the study participants' education level from the Longitudinal Integration Database for Health Insurance and Labor Market Studies at Statistics Sweden. The information was categorized as low (= primary school), middle (=upper secondary), or high (=post-secondary) education. The education level attained at 45 years of age was used. We retrieved information on country of birth from the same register. To be categorized as Swedish country of birth, we required both parents to be born in Sweden.

Data collection

We collected weight and height data from archived school healthcare records for all children born 1945 to 1968 attending schools in Gothenburg, Sweden. During this period, school was mandatory from 7 years of age, and the attendance to school healthcare was >98.5%.[1] Trained nurses measured weight and height at regular school healthcare visits, and the measurements were registered in school healthcare records. From 1947, a personal identity number (PIN) has been assigned to every Swedish resident, enabling linkage between the school healthcare data and nationwide registers. Weight and height measurements have also been retrieved from military conscription tests, mandatory until 2010 for all Swedish men.[2] To be eligible for the present study, a 10-digit PIN and a school healthcare record in the central archive were required.

Patient and public involvement

We have a continuous dialogue with the Swedish Obesity Association in the planning of our studies and as an important partner in communicating our study results to the public. The members have expressed a need for studies examining long-term consequences of overweight and obesity during childhood and adolescence as well as possible benefits of treatment.

© 2025 Olsson C et al et al. *JAMA Pediatrics*.

eResults. Supplemental Results

Changes in obesity status and percentile changes and the risk of CHD

There were 547 women (1.2%) and 283 men (0.5%) who had obesity in childhood, and 250 women (0.5%) and 534 men (0.9) who had obesity in young adult age. In less powered analyses, we evaluated the risk of CHD associated with childhood and young adult obesity. We found that childhood obesity with remission before young adulthood (i.e. non-obesity at young adult age, n=641) was not associated with significantly increased risk of CHD compared to individuals without obesity in both childhood and young adulthood (eFigure 2). Both the group who did not have obesity in childhood, but had obesity as young adults, and the group with obesity both in childhood and young adult age had increased risk of CHD events. However, the risk of CHD was not significantly different in individuals with pubertal onset obesity, compared to individuals with persistent obesity from childhood to young adulthood (eFigure 2). In the present study, only 80 women (0.2%) and 35 men (0.06%) had severe obesity in childhood, and nine women and seven men had severe obesity (above 40 kg/m^2) as young adults.

In addition, we performed analyses evaluating the CHD risk in individuals with a BMI in childhood and young adult age above the 95th percentile (eTable 7). We found no excess risk of a CHD event in individuals with a BMI in childhood above 95% and a young adult BMI within percentiles 15-85, compared with individuals with a childhood and young adult BMI within percentiles 15-85.

Risk of fatal coronary events

We next evaluated the risk of fatal CHD. Individuals with high BMI (above the 85th percentile) in childhood but with normal young adult BMI did not have an increased risk of fatal CHD compared to individuals with normal BMI in both childhood and young adulthood. By contrast, individuals with high BMI in young adulthood, regardless of childhood BMI status, had increased risk of fatal CHD compared to individuals with normal BMI in both childhood and young adulthood (eTable 12, eFigure 3). Sex-stratified analyses revealed similar results for men and women (eTable 12, eFigure 3).

Evaluation of competing risk

Fine- Gray models accounting for competing risks of non-CHD mortality revealed similar effect estimates (eTable 9).

Sensitivity analyses

We performed several sensitivity analyses. First, we excluded individuals with severe illness before the age of 22 years (i.e. cancer, diabetes and major surgery n=249; definitions see eTable 1). These exclusions did not alter the results (eTable 7). Secondly, we performed adjustments for diabetes. The results after adjustments were similar to the results without adjustment for diabetes (eTable 8).

Further, to evaluate temporal patterns of CHD events, we performed analyses of early (before 57.6 years

© 2025 Olsson C et al et al. *JAMA Pediatrics*.

of age) and late (after 57.6 years of age) CHD. These analyses showed slightly higher point estimates for early CHD events (eTable 10). In less powered exploratory analyses, we also divided the cases into before and after 44 years. These analyses revealed slightly more pronounced point estimates for events before than after 44 years of age (eTable 11).

eTable 1. Diagnostic Codes According to the *International Classification of Diseases* (*ICD*) System Defining the Study Outcome Coronary Heart Disease (CHD) in the Patient Register and Cause of Death Register in Sweden

	**ICD10**	**ICD9**	**ICD8**
**CHD events**[1]	I20-25	410-414	410-413
**Fatal CHD**[2]	I20-25	410-414	410-413
**Cancer**	C00-C97. D00-D48	140-239	140-239
**Diabetes**	E10-E14	250	250

1 Patient Register and Cause of Death Register

2 Cause of Death Register

© 2025 Olsson C et al et al. *JAMA Pediatrics*.

eTable 2. Characteristics of Individuals Who Were Not Included

	**Women and men**		**Women**		**Men**	
	Included (n=103,232)	Not included (n=35,981)	Included (n=45,965)	Not included (n=21,588)	Included (n=57,267)	Not included (n=14,393)
**Exposures**	Mean (SD)	Mean (SD)	Mean (SD)	Mean (SD)	Mean (SD)	Mean (SD)
Childhood[1] BMI	15.6 (1.5)	15.6 (1.5)*	15.5 (1.6)	15.5 (1.6)NS	15.7 (1.4)	15.8 (1.4)NS
Young adult[2] BMI	21.1 (2.5)	21.1 (2.5)NS	20.8 (2.5)	20.9 (2.5)*	21.4 (2.4)	21.3 (2.4)***
**Outcomes**	n (%)	n (%)	n (%)	n (%)	n (%)	n (%)
CHD events	5,736 (5.6)	1,944 (5.4)NS	1,298 (2.8)	838 (3.9)***	4,438 (7.7)	1,106 (7.7)NS
Fatal CHD	931 (0.9)	318 (0.9)NS	168 (0.4)	115 (0.5)***	763 (1.3)	203 (1.4)NS

Characteristics of the 35,981 individuals (women n=21,588, men n=14,393) born between 1945 and 1968 with complete personal identity number, who could not be included in the study due to missing childhood BMI or young adult BMI, or both, who were followed for a mean of 36.6 (SD 11.0) years after the age of 22 years. In total, 39,333 individuals were excluded due to missing one or both developmental BMI variables (eFigure 1). Of these, 3,352 individuals were censored before age 22 years. These individuals are not included in the table.

[1] Childhood BMI at 7 years of age for girls and 8 years of age for boys.

[2]Young adult BMI at 18 years of age for women and 20 years of age for men.

* $p<0.05$, ***$p<0.001$, NS= not significant vs included individuals

© 2025 Olsson C et al et al. *JAMA Pediatrics*.

eTable 3. Adjusted Hazard Ratios for Coronary Heart Disease (CHD) in Relation to Childhood and Young Adult Overweight, Including Obesity

	CHD events HR (95% CI)	***CHD events adjusted for socioeconomic status*** HR (95% CI)
Base model[1]		
**Full cohort**		
Childhood overweight	**1.15 (1.02; 1.28)**	**1.16 (1.03; 1.30)**
Young adult overweight	**1.71 (1.56; 1.86)**	**1.63 (1.50; 1.79)**
**Women**		
Childhood overweight	1.11 (0.91; 1.35)	1.12 (0.92; 1.36)
Young adult overweight	**1.96 (1.60; 2.40)**	**1.83 (1.49; 2.24)**
**Men**		
Childhood overweight	**1.17 (1.01; 1.34)**	**1.18 (1.03; 1.36)**
Young adult overweight	**1.66 (1.50; 1.83)**	**1.60 (1.45; 1.76)**
Adjusted model[2]		
**Full cohort**		
Childhood overweight	0.91 (0.80; 1.02)	0.91 (0.80; 1.02)
Young adult overweight	**1.75 (1.59; 1.93)**	**1.75 (1.59; 1.93)**
**Women**		
Childhood overweight	0.88 (0.71; 1.09)	0.88 (0.71; 1.09)
Young adult overweight	**2.05 (1.64; 2.57)**	**2.05 (1.64; 2.57)**
**Men**		
Childhood overweight	0.91 (0.78; 1.06)	0.91 (0.78; 1.06)
Young adult overweight	**1.70 (1.53; 1.89)**	**1.70 (1.53; 1.89)**

Hazard ratios (HR) calculated using Cox proportional hazards regression with subjects with normal weight used as reference. Total cohort including 103,232 women and men born between 1945 and 1968 (45,965 women and 57,267 men), followed for a mean of 37.6 (SD 10.7) years after the age of 22 years.

[1]The base model is adjusted for birth year and country of birth, and for the full cohort additionally adjusted for sex.

[2]The adjusted model includes both childhood overweight and young adult overweight and is adjusted for birth year and country of birth, and for the full cohort additionally adjusted for sex. The right column is adjusted for socioeconomic status, defined as educational attainment at 45 years of age Childhood overweight including obesity is defined based on International Obesity Task Force (IOTF) cut-offs at 7 and 8 years of age (girls and boys, respectively).[12] Young adult overweight including obesity at 18 and 20 years of age (women and men, respectively) is based on the cut-off 25 kg/m^2 for both sexes. Overweight incl obesity in girls n=3,981, in boys n=2,492, in young adult women n=2,350, in young adult men n=4,417. CHD events in women n=1,298, in men n=4,438

CI=confidence interval.

© 2025 Olsson C et al et al. *JAMA Pediatrics*.

eTable 4. Absolute Risks and Adjusted Hazard Ratios for Coronary Heart Disease in Relation to Changes in Weight Status Between Childhood and Early Adulthood

		Risk of Coronary Heart Disease			***Adjusted for socioeconomic status***
	N in group	***Cases***	***AR***	**HR (95% CI)**	**HR (95% CI)**
**Full cohort (childhood/young adulthood)**					
Normal weight/normal weight	92,374	4,997	5.4%	*Reference*	*Reference*
Overweight/normal weight	4,091	165	4.0%	0.98 (0.84; 1.14)	1.02 (0.87; 1.19)
Normal weight/overweight	4,385	413	9.4%	**1.83 (1.66; 2.03)**	**1.75 (1.59; 1.94)**
Overweight/overweight	2,382	161	6.8%	**1.53 (1.30; 1.78)**	**1.47 (1.26; 1.72)**
**Women**					
Normal weight/normal weight	40,796	1,128	2.8%	*Reference*	*Reference*
Overweight/normal weight	2,819	65	2.3%	0.87 (0.68; 1.12)	0.89(0.69; 1.15)
Normal weight/overweight	1,188	54	4.5%	**2.05 (1.56; 2.70)**	**1.90 (1.45; 2.50)**
Overweight/overweight	1,162	51	4.4%	**1.90 (1.44; 2.52)**	**1.80 (1.36; 2.38)**
**Men**					
Normal weight/normal weight	51,578	3,869	7.5%	*Reference*	*Reference*
Overweight/normal weight	1,272	100	7.9%	1.07 (0.88; 1.31)	1.12 (0.92; 1.37)
Normal weight/overweight	3,197	359	11.2%	**1.80 (1.62; 2.01)**	**1.74 (1.56; 1.93)**
Overweight/overweight	1,220	110	9.0%	**1.40 (1.15; 1.69)**	**1.36 (1.12; 1.64)**

Hazard ratios (HR) calculated using Cox proportional hazards regression, adjusted for birth year and country of birth, for the full cohort additionally adjusted for sex, and for the right column additionally adjusted for education level at 45 years of age. Total cohort including 103,232 women and men born between 1945 and 1968 (45,965 women and 57,267 men), followed for a mean of 37.6 (SD 10.7) years after the age of 22 years. Childhood overweight is defined based on International Obesity Task Force (IOTF) cut-offs at 7 and 8 years of age (girls and boys, respectively). Young adult overweight at 18 and 20 years of age (women and men, respectively) is based on the cut-off 25 kg/m^2 for both sexes. Socioeconomic status defined as educational attainment at 45 years of age. Overweight includes obesity, AR=absolute risk, CI=confidence interval.

© 2025 Olsson C et al et al. *JAMA Pediatrics*.

eTable 5. BMI in Childhood and Young Adulthood for Groups Categorized According to Normal Weight and Overweight Status in Childhood and Young Adult Age

**Women**	**Childhood BMI**	**Young adult BMI**	**The pubertal BMI change**
Normal weight/normal weight	15.2 (1.12)	20.3 (1.83)	5.1 (1.56)
Overweight/normal weight	18.8 (1.11)	22.3 (1.66)	3.5 (1.89)
Normal weight/overweight	16.4 (0.89)	26.7 (1.82)	10.2 (1.97)
Overweight/overweight	19.6 (1.73)	27.8 (2.90)	8.2 (2.83)
**Men**			
Normal weight/normal weight	15.5 (1.07)	20.9 (1.84)	5.4 (1.56)
Overweight/normal weight	19.5 (1.09)	22.9 (1.42)	3.4 (1.79)
Normal weight/overweight	16.6 (1.02)	26.9 (1.92)	10.2 (2.08)
Overweight/overweight	20.2 (1.76)	28.5 (3.07)	8.3 (2.96)

BMI in childhood and young adulthood for groups categorized according to normal weight and overweight status in childhood and young adult age. The study cohort includes 103,232 women and men born between 1945 and 1968 (45,965 women and 57,267 men), followed for a mean of 37.6 (SD 10.7) years after the age of 22 years. Childhood overweight is defined based on International Obesity Task Force (IOTF) cut-offs at 7 and 8 years of age (girls and boys, respectively). Young adult overweight at 18 and 20 years of age (women and men, respectively) is based on the cut-off 25 kg/m^2 for both sexes. Overweight includes obesity.

© 2025 Olsson C et al et al. *JAMA Pediatrics*.

eTable 6. Absolute Risks and Adjusted Hazard Ratios for Coronary Heart Disease in Relation to Changes in Weight Status Between Childhood and Early Adulthood in Individuals With Both Parents Born in Sweden

		Risk of Coronary Heart Disease			***Adjusted for socioeconomic status***
	N in group	***Cases***	***AR***	**HR (95% CI)**	**HR (95% CI)**
**Full cohort (childhood/young adulthood)**					
Normal weight/normal weight	79,736	4,408	5.5%	*Reference*	*Reference*
Overweight/normal weight	3,555	145	4.1%	0.97 (0.82; 1.14)	1.00 (0.85; 1.19)
Normal weight/overweight	3,677	352	9.6%	**1.81 (1.63; 2.02)**	**1.73 (1.55; 1.93)**
Overweight/overweight	2,018	144	7.1%	**1.56 (1.32; 1.84)**	**1.50 (1.27; 1.77)**
**Women**					
Normal weight/normal weight	35,065	987	2.8%	*Reference*	*Reference*
Overweight/normal weight	2,444	59	2.4%	0.89 (0.69; 1.16)	0.92 (0.70; 1.19)
Normal weight/overweight	980	47	4.8%	**2.10 (1.57; 2.81)**	**1.93 (1.44; 2.59)**
Overweight/overweight	958	45	4.7%	**2.00 (1.48; 2.69)**	**1.92 (1.42; 2.59)**
**Men**					
Normal weight/normal weight	44,671	3,421	7.7%	*Reference*	*Reference*
Overweight/normal weight	1,111	86	7.7%	1.03 (0.83; 1.28)	1.08 (0.87; 1.34)
Normal weight/overweight	2,697	305	11.3%	**1.77 (1.58; 2.00)**	**1.70 (1.52; 1.92)**
Overweight/overweight	1,060	99	9.3%	**1.41 (1.16; 1.73)**	**1.37 (1.12; 1.67)**

Hazard ratios (HR) calculated using Cox proportional regression, adjusted for birth year, for the full cohort additionally adjusted for sex, and for the right column additionally adjusted for socioeconomic status defined as educational attainment at 45 years of age. A total number of 88,986 women and men born between 1945 and 1968 (39,447 women and 49,539 men), born in Sweden and with parents born in Sweden, were followed for a mean of 37.6 (SD 10.7) years after the age of 22 years. Childhood overweight is defined based on International Obesity Task Force (IOTF) cut offs at 7 and 8 years of age (girls and boys, respectively). Young adult overweight at 18 and 20 years of age (women and men, respectively) is based on the cutoff 25 kg/m^2 for both sexes. nw normal weight, ow overweight including obesity, AR absolute risk, CI confidence interval.

© 2025 Olsson C et al et al. *JAMA Pediatrics*.

eTable 7. Absolute Risks and Adjusted Hazard Ratios for Coronary Heart Disease in Relation to Changes in Weight Status Between Childhood and Early Adulthood, After Exclusion of Individuals With Severe Disease or Major Surgery Before 22 Years of Age

		Risk of Coronary Heart Disease			***Adjusted for socioeconomic status***
	N in group	***Cases***	***AR***	**HR (95% CI)**	**HR (95% CI)**
**Full cohort (childhood/young adulthood)**					
Normal weight/normal weight	92,161	4,966	5.4%	*Reference*	*Reference*
Overweight/normal weight	4,073	164	4.0%	0.98 (0.84; 1.15)	1.02 (0.87; 1.20)
Normal weight/overweight	4,374	412	9.4%	**1.84 (1.67; 2.04)**	**1.76 (1.59; 1.95)**
Overweight/overweight	2,375	159	6.7%	**1.52 (1.30; 1.78)**	**1.47 (1.25; 1.72)**
**Women**					
Normal weight/normal weight	40,664	1,111	2.7%	*Reference*	*Reference*
Overweight/normal weight	2,804	65	2.3%	0.89 (0.69; 1.14)	0.91 (0.71; 1.17)
Normal weight/overweight	1,182	54	4.6%	**2.09 (1.59; 2.75)**	**1.94 (1.47; 2.55)**
Overweight/overweight	1,156	49	4.2%	**1.87 (1.40; 2.49)**	**1.76 (1.32; 2.34)**
**Men**					
Normal weight/normal weight	51,497	3,855	7.5%	*Reference*	*Reference*
Overweight/normal weight	1,269	99	7.8%	1.07 (0.87; 1.30)	1.12 (0.92; 1.36)
Normal weight/overweight	3,192	358	11.2%	**1.81 (1.62; 2.01)**	**1.74 (1.56; 1.94)**
Overweight/overweight	1,219	110	9.0%	**1.40 (1.16; 1.69)**	**1.36 (1.13; 1.65)**

Hazard ratios (HR) calculated using Cox proportional hazards regression, adjusted for birth year and country of birth. The full cohort is additionally adjusted for sex, and the right column additionally adjusted for socioeconomic status defined as educational attainment at 45 years of age. We have excluded individuals with severe disease defined as cancer, diabetes or surgery to the heart and vessels orthopaedic procedures before age 22 years (n=249). Childhood overweight including obesity is defined based on International Obesity Task Force (IOTF) cut-offs at 7 and 8 years of age (girls and boys, respectively). Young adult overweight including obesity at 18 and 20 years of age (women and men, respectively) is based on the cut-off 25 kg/m^2 for both sexes. Overweight includes obesity, AR=absolute risk, CI=confidence interval.

© 2025 Olsson C et al et al. *JAMA Pediatrics*.

eTable 8. Adjusted Hazard Ratios for Coronary Heart Disease in Relation to Changes in Weight Status Between Childhood and Early Adulthood, With Adjustment for Diabetes Registered Before Event or Censoring

	Risk of Coronary Heart Disease	*Risk of Coronary Heart Disease adj. for socioeconomic status*
	**HR (95% CI)**	**HR (95% CI)**
**Full cohort (childhood/young adulthood)**		
Normal weight/normal weight	*Reference*	*Reference*
Overweight/normal weight	0.97 (0.83; 1.14)	1.01 (0.87; 1.18)
Normal weight/overweight	**1.78 (1.61; 1.97)**	**1.71 (1.55; 1.90)**
Overweight/overweight	**1.47 (1.26; 1.73)**	**1.43 (1.22; 1.68)**
**Women**		
Normal weight/normal weight	*Reference*	*Reference*
Overweight/normal weight	0.85 (0.66; 1.09)	0.87 (0.68; 1.12)
Normal weight/overweight	**1.82 (1.39; 2.40)**	**1.70 (1.29; 2.24)**
Overweight/overweight	**1.71 (1.29; 2.27)**	**1.63 (1.23; 2.17)**
**Men**		
Normal weight/normal weight	*Reference*	*Reference*
Overweight/normal weight	1.07 (0.88; 1.30)	**1.12 (0.92; 1.37)**
Normal weight/overweight	**1.79 (1.60; 1.99)**	**1.73 (1.55; 1.93)**
Overweight/overweight	**1.38 (1.14; 1.67)**	**1.35 (1.12; 1.63)**

Hazard ratios (HR) for coronary heart disease (CHD) calculated using Cox proportional hazards regression, adjusted for birth year, country of birth, and diabetes diagnoses registered before event or censoring. The analyses in the full cohort were additionally adjusted for sex. The right column is also adjusted for socioeconomic status defined as educational attainment at 45 years of age. Childhood overweight is defined based on International Obesity Task Force (IOTF) cut-offs at 7 and 8 years of age (girls and boys, respectively). Young adult overweight at 18 and 20 years of age is based on the cut-off 25 kg/m^2 for both sexes. Overweight includes obesity. AR=absolute risk, CI=confidence interval.

© 2025 Olsson C et al et al. *JAMA Pediatrics*.

eTable 9. Adjusted Hazard Ratios for Coronary Heart Disease in Relation to Changes in Weight Status Between Childhood and Early Adulthood, Accounting for Competing Risks of non-CHD Mortality Using the Fine and Gray Model

**Full cohort (childhood/young adulthood)**	***Risk of Coronary Heart Disease***	***Risk of Coronary Heart Disease adjusted for socioeconomic status***
Normal weight/normal weight	*Reference*	*Reference*
Overweight/normal weight	0.98 (0.84; 1.15)	1.01 (0.86; 1.18)
Normal weight/overweight	**1.81 (1.64; 2.00)**	**1.73 (1.57; 1.92)**
Overweight/overweight	**1.49 (1.27; 1.74)**	**1.45 (1.24; 1.70)**

Hazard ratios (HR) and 95% confidence interval (CI) calculated using the Fine and Gray model, accounting for competing risks of non-CHD mortality and adjusted for birth year, country of birth and sex. The models in the right column are additionally adjusted for socioeconomic status defined as educational attainment at 45 years of age. Childhood overweight is defined based on International Obesity Task Force (IOTF) cut-offs at 7 and 8 years of age (girls and boys, respectively). Young adult overweight at 18 and 20 years of age (women and men, respectively) is based on the cut-off 25 kg/m^2 for both sexes. Overweight includes obesity.

© 2025 Olsson C et al et al. *JAMA Pediatrics*.

eTable 10. Adjusted Hazard Ratios for Early (< 57.6) and Late (>57.6) Coronary Heart Disease in Relation to Changes in Weight Status Between Childhood and Early Adulthood

	Risk of Early Coronary Heart Disease	***Risk of Late Coronary Heart Disease***
	**HR (95% CI)**	**HR (95% CI)**
**Full cohort (childhood/young adulthood)**		
Normal weight/normal weight	*Reference*	*Reference*
Overweight/normal weight	1.05 (0.85; 1.30)	0.91 (0.72; 1.14)
Normal weight/overweight	**2.04 (1.79; 2.33)**	**1.59 (1.35; 1.87)**
Overweight/overweight	**1.81 (1.49; 2.21)**	1.19 (0.92; 1.55)
**Women**		
Normal weight/normal weight	*Reference*	*Reference*
Overweight/normal weight	0.88 (0.62; 1.25)	0.86 (0.60; 1.23)
Normal weight/overweight	**2.08 (1.45; 2.99)**	**2.01 (1.33; 3.06)**
Overweight/overweight	**2.41 (1.72; 3.37)**	1.27 (0.76; 2.13)
**Men**		
Normal weight/normal weight	*Reference*	*Reference*
Overweight/normal weight	1.19 (0.91; 1.55)	0.96 (0.71; 1.29)
Normal weight/overweight	**2.04 (1.77; 2.34)**	**1.53 (1.29; 1.82)**
Overweight/overweight	**1.60 (1.25; 2.04)**	1.17 (0.86; 1.58)

Hazard ratios (HR) for early (before 57.6 years) and late (after 57.6 years) coronary heart disease (CHD), calculated using Cox proportional hazards regression, adjusted for birth year and country of birth, for the full cohort additionally adjusted for sex. Childhood overweight is defined based on International Obesity Task Force (IOTF) cut-offs at 7 and 8 years of age (girls and boys, respectively). Young adult overweight at 18 and 20 years of age (women and men, respectively) is based on the cut-off 25 kg/m^2 for both sexes. Overweight includes obesity. AR=absolute risk, CI=confidence interval.

© 2025 Olsson C et al et al. *JAMA Pediatrics*.

eTable 11. Adjusted Hazard Ratios for Coronary Heart Disease Before and After 44 Years of Age, in Relation to Changes in Weight Status Between Childhood and Early Adulthood

	Risk of Coronary Heart Disease before 44 years of age		***Risk of Coronary Heart Disease after 44 years of age***	
	Cases/N in group	**HR (95% CI)**	Cases/N in group	**HR (95% CI)**
**Full cohort (childhood/young adulthood)**				
Normal weight/normal weight	300/92,374	*Reference*	4,697/85,030	*Reference*
Overweight/normal weight	15/4,091	1.43 (0.85; 2.40)	150/3,791	0.95 (0.81; 1.12)
Normal weight/overweight	36/4,385	**2.20 (1.56; 3.12)**	377/4,070	**1.81 (1.63; 2.01)**
Overweight/overweight	24/2,382	**3.24 (2.14; 4.91)**	137/2,212	**1.40 (1.18; 1.66)**

Hazard ratios (HR) for coronary heart disease (CHD) occurring before and after 44 years of age, calculated using Cox proportional hazards regression, adjusted for birth year and country of birth, for the full cohort additionally adjusted for sex. Childhood overweight is defined based on International Obesity Task Force (IOTF) cut-offs at 7 and 8 years of age (girls and boys, respectively). Young adult overweight at 18 and 20 years of age (women and men, respectively) is based on the cut-off 25 kg/m^2 for both sexes. Overweight includes obesity. AR=absolute risk, CI=confidence interval.

© 2025 Olsson C et al et al. *JAMA Pediatrics*.

eTable 12. Absolute Risks and Adjusted Hazard Ratios for Coronary Heart Disease (CHD) Events and Fatal CHD, in Relation to Normal BMI (Under 85th Percentile) or High BMI (Over 85th Percentile)

		CHD events			*Fatal CHD*		
	N in group	***Cases***	***AR***	**HR (95% CI)**	***Cases***	***AR***	**HR (95% CI)**
**Full cohort (childhood/young adulthood)**							
Normal BMI/normal BMI	79,648	4,258	5.3%	*Reference*	647	0.8%	*Reference*
High BMI/normal BMI	8,099	424	5.2%	0.99 (0.90; 1.10)	79	1.0%	**1.34 (1.06; 1.70)**
Normal BMI/high BMI	8,099	577	7.1%	**1.58 (1.45; 1.72)**	104	1.3%	**1.73 (1.40; 2.13)**
High BMI/high BMI	7,386	477	6.5%	**1.41 (1.29; 1.55)**	101	1.4%	**1.95 (1.58; 2.41)**
**Women**							
Normal BMI/normal BMI	35,500	974	2.7%	*Reference*	115	0.3%	*Reference*
High BMI/normal BMI	3,570	98	2.7%	1.00 (0.81; 1.23)	13	0.4%	1.23 (0.69; 2.18)
Normal BMI/high BMI	3,570	121	3.4%	**1.50 (1.24; 1.81)**	20	0.6%	**1.88 (1.16; 3.03)**
High BMI/high BMI	3,325	105	3.2%	**1.32 (1.08; 1.62)**	20	0.6%	**1.99 (1.23; 3.21)**
**Men**							
Normal BMI/normal BMI	44,148	3,284	7.4%	*Reference*	532	1.2%	*Reference*
High BMI/normal BMI	4,529	326	7.2%	0.99 (0.88; 1.11)	66	1.5%	**1.36 (1.05; 1.76)**
Normal BMI/high BMI	4,529	456	10.1%	**1.60 (1.45; 1.77)**	84	1.9%	**1.69 (1.34; 2.13)**
High BMI/high BMI	4,061	372	9.2%	**1.44 (1.29; 1.60)**	81	2.0%	**1.92 (1.52; 2.43)**

Hazard ratios (HR) were calculated using Cox proportional hazards regression adjusted for birth year and country of birth, and for the full cohort additionally adjusted for sex. Full cohort including 103,232 women and men born between 1945 and 1968 (45,965 women and 57,267 men), followed for a mean of 37.6 (SD 10.7) years after the age of 22 years. Normal BMI was defined as below 85th percentile. High BMI in childhood was defined as BMI above the 85th percentile for age and sex in childhood (7 years for girls and 8 years for boys). Percentiles were made per sex, internal to this cohort. High BMI in adulthood was defined as BMI above the 85th percentile for age and sex in young adulthood (18 years for women and 20 years for men) respectively. Percentiles were made per sex, internal to this cohort. AR=absolute risk, HR=hazard ratio, CI=confidence interval

© 2025 Olsson C et al et al. *JAMA Pediatrics*.

eTable 13. Adjusted Hazard Ratios for Coronary Heart Disease (CHD) Events in Relation to Sex-Specific BMI 95th Percentile in Childhood by Sex-Specific BMI 95th Percentile in Young Adulthood and the Risk of Coronary Heart Disease in Women and Men

**BMI percentile in childhood**	**BMI percentile in young adulthood**	
	**15th-85th**	**>95th**
	**Hazard ratio for coronary heart disease (95% CI)** n / cases	
**Full cohort**		
**< 15th**	1.07 (0.97; 1.19)<br>8061 / 422	**2.96 (1.48; 5.92)**<br>54 / 8
**15th-85th**	*Reference*<br>56,274 / 2952	**2.12 (1.82; 2.49)**<br>1814 / 165
**>95th**	0.96 (0.78; 1.19)<br>1792 / 91	**1.62 (1.36; 1.92)**<br>1858 / 135
**Women**		
**< 15th**	**1.27 (1.04; 1.56)**<br>3638 / 112	**-**<br>19 / 2
**15th-85th**	*Reference*<br>25,068 / 656	**2.36 (1.69; 3.29)**<br>770 / 37
**>95th**	0.92 (0.58; 1.45)<br>810 / 19	**2.18 (1.58; 3.01)**<br>817 / 39
**Men**		
**< 15th**	1.01 (0.90; 1.14)<br>4423 / 310	**2.61 (1.17; 5.81)**<br>35 / 6
**15th-85th**	*Reference*<br>31,206 / 2296	**2.06 (1.72; 2.46)**<br>1044 / 128
**>95th**	0.97 (0.77; 1.23)<br>982 / 72	**1.46 (1.19; 1.80)**<br>1041 / 96

Hazard ratios (HR) calculated using Cox proportional hazards regression with adjustment for birth year and country of birth, and for the full cohort additionally adjusted for sex. In groups with fewer than five cases of CHD, no HR was calculated. Childhood BMI was calculated at 7 and 8 years of age for girls and boys, respectively, and young adult BMI at 18 and 20 years of age for women and men, respectively. Percentiles were calculated internal to this cohort.

© 2025 Olsson C et al et al. *JAMA Pediatrics*.

eFigure 1. Flowchart of Included Individuals in the Study

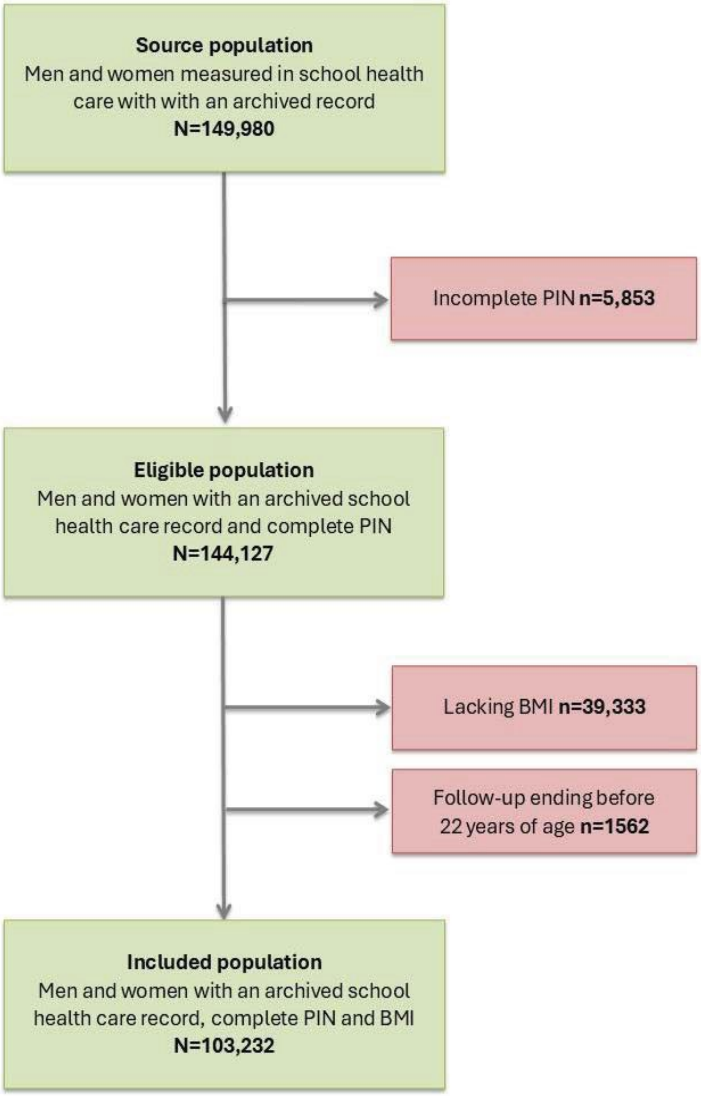

PIN, personal identity number, BMI, body mass index

© 2025 Olsson C et al et al. *JAMA Pediatrics*.

eFigure 2. Risk of CHD Events and Fatal CHD According to BMI Above the 85th Percentile, in Childhood and Young Adulthood

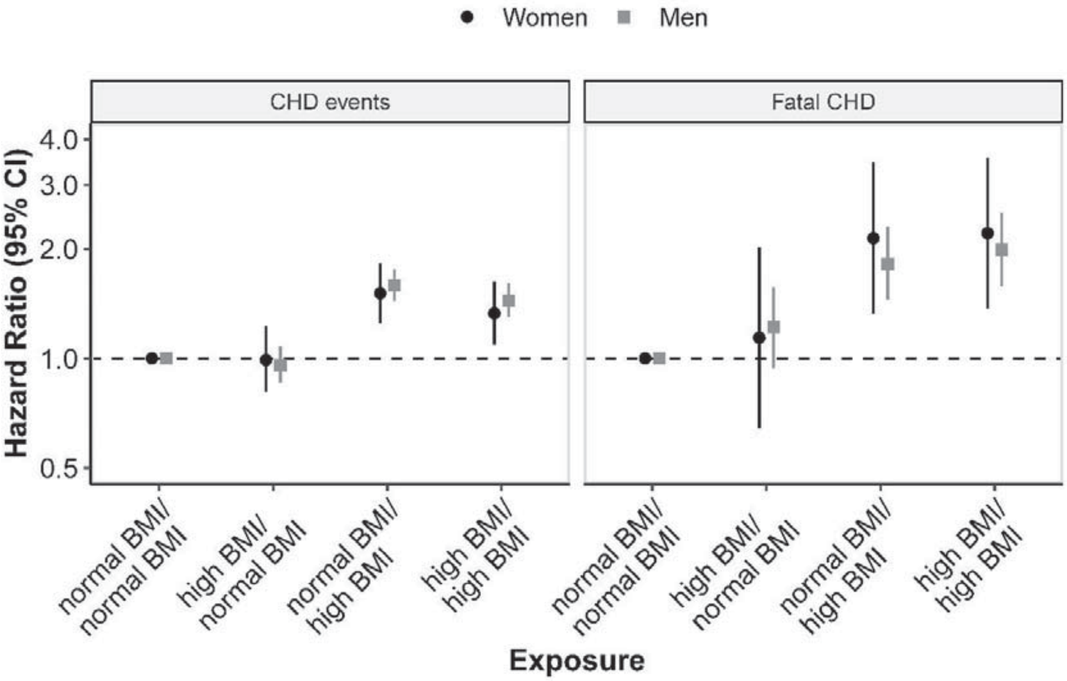

Hazard ratios estimated using Cox proportional hazards regression models with 95% confidence intervals (CI) adjusted for birth year and country of birth. High BMI in childhood and young adulthood defined as a BMI above the 85th percentile for age and sex (childhood 7 and 8 years for girls and boys, young adulthood 18 and 20 years for women and men) in the present cohort. Individuals with normal BMI in childhood and young adulthood were used as the reference group.

© 2025 Olsson C et al et al. *JAMA Pediatrics*.

eFigure 3. Risk of CHD Events According to Obesity in Childhood and Young Adulthood

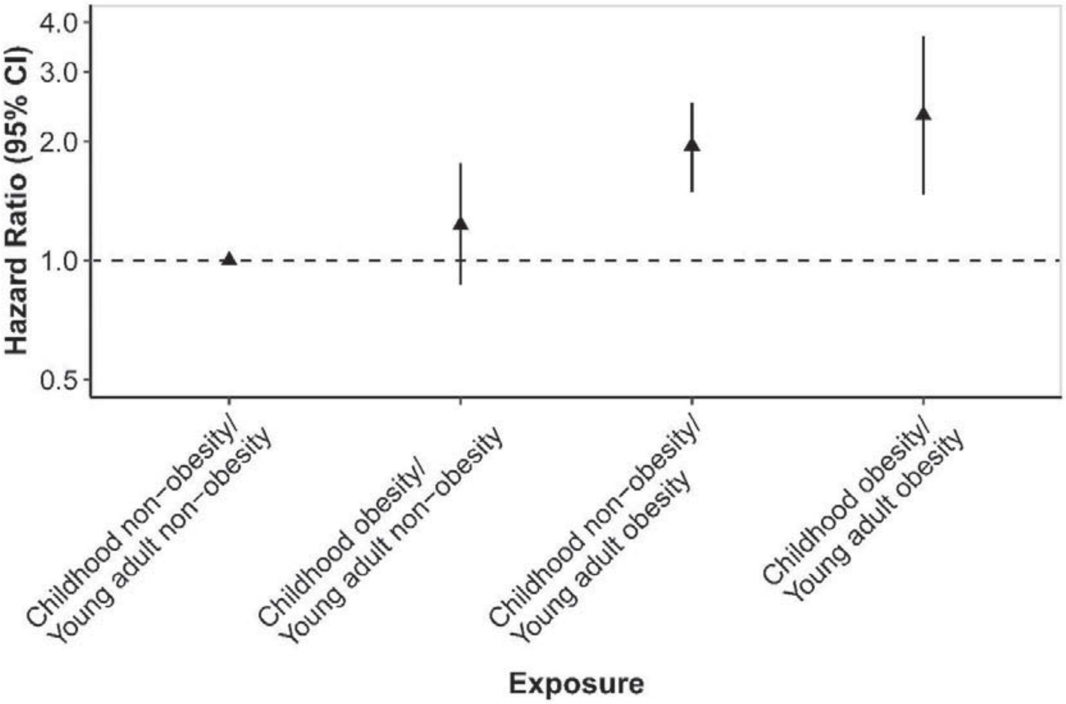

Hazard ratios estimated using Cox proportional hazards regression models with 95% confidence intervals (CI). Obesity in childhood was defined according to the IOTF cut-offs for age and sex (at 7 years for girls and 8 years for boys), and obesity in young adulthood (at 18 years for women and 20 years for men) was defined as a BMI above 30 kg/m2. Individuals without obesity in childhood or young adulthood were used as the reference group, men and women analyzed together.

© 2025 Olsson C et al et al. *JAMA Pediatrics*.

eReferences.

1. Herlitz CW. *Skolhygienens historia*. Bergvalls; 1961.

2. Pliktverket. Vår historia. Accessed 2023-05-17, https://www.pliktverket.se/om-myndigheten/var-historia

3. Fine JP, Gray RJ. A Proportional Hazards Model for the Subdistribution of a Competing Risk. *Journal of the American Statistical Association*. 1999/06/011999;94(446):496-509. doi:10.1080/01621459.1999.10474144

© 2025 Olsson C et al et al. *JAMA Pediatrics*.
