# Supplementary material for: Change in Weight Status From Childhood to Young Adulthood and Risk of Adult Coronary Heart Disease
Source: JAMA Pediatr. 2025 Dec 1;180(2):179–86. doi: 10.1001/jamapediatrics.2025.4950 (PMC12670265; doi:10.1001/jamapediatrics.2025.4950)
Supplement: Supplement 2. — Data Sharing Statement [file jamapediatr-e254950-s002.pdf]

Data Sharing Statement

Ohlsson. Change in Weight Status From Childhood to Young Adulthood and Risk of Adult Coronary Heart Disease. *JAMA Pediatr*. Published December 01, 2025.
doi:10.1001/jamapediatrics.2025.4950

Data
Data available: No

Additional Information
Explanation for why data not available: The data that support the findings of this study are available from the corresponding author upon reasonable request and upon approval from the University of Gothenburg according to mandatory national law but are not publicly available due to privacy and ethical restrictions.
